# Changes in Stress and Appetite Responses in Male Power-Trained Athletes during Intensive Training Camp

**DOI:** 10.3390/nu9080912

**Published:** 2017-08-21

**Authors:** Satomi Oshima, Chisato Takehata, Ikuko Sasahara, Eunjae Lee, Takao Akama, Motoko Taguchi

**Affiliations:** 1 Waseda Institute of Sports Nutrition, Waseda University, 2-579-15, Mikajima, Tokorozawa, Saitama 359-1192, Japan; msatomi3939@yahoo.co.jp; 2 Graduate School of Sport Sciences, Waseda University, 2-579-15, Mikajima, Tokorozawa, Saitama 359-1192, Japan; takehatachisato@gmail.com; 3 Institute of Food Sciences & Technologies, Ajinomoto co., Inc. 1-1, Suzuki-cho, Kawasaki-ku, Kawasaki, Kanagawa 210-8681, Japan; ikuko_sasahara@ajinomoto.com; 4 Waseda Institute for Sport Sciences, Waseda Univerity, 2-579-15, Mikajima, Tokorozawa, Saitama 359-1192, Japan; eunjaelee0421@gmail.com; 5 Faculty of Sport Sciences, Waseda University, 2-579-15, Mikajima, Tokorozawa, Saitama 359-1192, Japan; takao-akama@waseda.jp

**Keywords:** ghrelin, cortisol, salivary IgA, POMS, session RPE

## Abstract

An intensive consecutive high-volume training camp may induce appetite loss in athletes. Therefore, this study aimed to investigate the changes in stress and appetite responses in male power-trained athletes during an intensive training camp. The measurements at Day 2 and at the end of a 9-day intensive training camp (Camp1 and Camp2, respectively) were compared with those of the resting period (Rest) and the regular training period (Regular; *n* = 13). The stress state was assessed based on plasma cortisol level, salivary immunoglobulin A level, and a profile of mood states score. The sensation of appetite was assessed using visual analog scale scores, and fasting plasma acylated ghrelin, insulin, and glucose were measured. The cortisol concentrations were significantly higher at Camp2 (466.7 ± 60.7 nmol∙L^−1^) than at Rest (356.3 ± 100.9 nmol∙L^−1^; *p* = 0.002) or Regular (361.7 ± 111.4 nmol∙L^−1^; *p* = 0.003). Both prospective and actual food consumption significantly decreased at Camp2, and acylated ghrelin concentration was significantly lower at Camp1 (34.2 ± 8.0 pg∙mL^−1^) and Camp2 (32.0 ± 8.7 pg∙mL^−1^) than at Rest (47.2 ± 11.2 pg∙mL^−1^) or Regular (53.4 ± 12.6 pg∙mL^−1^). Furthermore, the change in acylated ghrelin level was negatively correlated with the change in cortisol concentration. This study’s findings suggest that an early-phase physiological stress response may decrease the acylated ghrelin level in male power-trained athletes during an intensive training camp.

## 1. Introduction

Competitive athletes often suffer from a loss of appetite when under continual excessive training. Since loss of appetite is a known symptom of overtraining, prolonged high-volume daily training seems to induce a physiological disturbance in the appetite regulatory system [1,2]. Depending on the severity, a loss of appetite in athletes may interfere with adequate energy and nutrient intake, and result in inadequate recovery [3]. Overtraining is an accumulation of training and/or non-training stress, which results in a long-term decrease in performance capacity that takes several months to develop. However, over-reaching (non-functional) induces a short-term decrease in performance capacity, and the initial signs of over-reaching may become apparent after only a few weeks of hard training [4]. This may have important implications for athletes participating in a short-duration high-volume intensive training camp. To avoid developing over-reaching or overtraining syndrome, it is important that athletes understand the correlation between change in appetite and early signs of training stress.

When athletes are under stress from excessive daily training, changes in hormones, immunological parameters, and mood states are observed [5,6]. Cortisol is a primary stress hormone that is released from the pituitary gland under sympathetic activation [5]. Increased resting cortisol may indicate acutely elevated physiological stress, while decreased resting cortisol is a sign of more chronic stress as seen in overtraining syndrome [6]. Immune function and psychological state are also disturbed by excessive training stress [1,5]. Several studies have reported that salivary immunoglobulin A (SIgA) level decreases in intensive training interventions [7,8].

Appetite is known to be influenced by exercise training and different forms of stress in addition to energy status [9]. Ghrelin is an appetite stimulating hormone that is predominantly released from the gastric cells within the stomach, and is the only gut hormone known to enhance appetite [10,11]. Acylated ghrelin, the activated form of ghrelin, appears to closely affect appetite sensation since it can cross the blood–brain barrier [12]. Broom et al. and many others have reported decreases in acylated ghrelin levels and appetite after higher-intensity endurance and strengthening exercises [13,14,15].

Many studies have investigated the signs and symptoms of training stress in athletes during excessive training periods [16,17,18]. However, only a few studies have assessed appetite changes in the context of changes in subjective sensations and regulatory hormones during intensive training periods [19]. Hoffman et al. examined biochemical changes for possible indicators of early-stage overtraining in elite basketball players during a 4-week training camp [16]. There was a significant increase in cortisol concentration after 100–145 min of training per day of consecutive training; however, no significant change in subjective appetite sensation was reported. Unfortunately, appetite regulatory hormones were not measured in that study. Therefore, the purpose of this study was to investigate the changes in stress and appetite responses in male power-trained athletes during an intensive training camp. We hypothesized that male power-trained athletes would accumulate training stress that would lead to appetite loss, including changes in appetite regulatory hormones, during an intensive training camp.

## 2. Materials and Methods

### 2.1. Participants

The subjects were 13 Japanese college male athletes who were 18–22 years of age. All are members of the American football team in the East Japan College Football Association division 1. To narrow the range of body sizes and physical characteristics, subjects were limited to backs and skilled positions (e.g., wide receivers, linebackers, defensive backs, and quarterbacks). All subjects had >4 years of training experience playing American football. Athletes were regularly trained at the home field located on campus. None of the subjects had been taking any medications or smoking habitually. Subjects were given oral and written descriptions of the study and were free to withdraw at any time. Informed consent was obtained prior to the study’s initiation. The study was approved by the Human Research Committee of Waseda University for the inclusion of human subjects according to the Declaration of Helsinki (ethical approval code: 2013-217; 7 November 2013).

### 2.2. Study Design

This study was designed to examine the changes in biochemical and psychological indicators of stress and appetite during a training camp. The measurements were compared between the start and end of the training camp with the rest or regular training period. The intensive training camp lasted for 9 days, and the athletes trained every day without any period of rest. The measurements were performed during the summer training camp where the camp is held every year. The first measurement of the camp (Camp1) was collected in the early morning on the 2nd day of camp, while the second measurement of the camp (Camp2) was collected at the same time on the 9th day of camp. Most of the measurements of the regular training period (Regular) were collected for 2 days in the same week during the off-season, except for exercise volume measurements that were collected for 3 days. The exercise volume measurements, which were collected for 3 days, were then averaged to reduce bias due to the difference in training menu by day during the regular training period. In addition, approximately 2 weeks prior to the training camp, the subjects stopped undergoing daily training for 2 days. The assessments were taken the following morning as rest period measurements (Rest). All of the parameters except for exercise volume were collected in the early morning; specifically, the blood samples were collected approximately from 06:00 to 07:00.

During the training camp, there were two training sessions per day from roughly 08:00 to 11:00 and from 14:00 to 17:00. The training flow and approximate time for the morning session were the following; warming up (10 min), fundamental training (20 min), position-group training (30 min), team training (120 min), position-group training (20 min), and cooling down (10 min). The training flow and approximate time for the afternoon session were the following; warming up (10 min), fundamental training (20 min), position-group training (60 min), team training (60 min), circuit training (30 min), run training (15 min), and cooling down (10 min). Warming up mainly consisted of dynamic movements and stretching. Fundamental training included short and repetitive position-specific movements, such as tackles and ball handlings while wearing protective equipment. The position-specific training varied according to the task of each position. During team training, a set of game style movements was repeated, with each set lasting for only 15–30 s. The circuit training was for improving basic physical fitness, including agility, power, and speed. The run training included short to middle distance interval training. The training menu of Day 5 was resistance training only for 2–3 h in the fitness room. Regular daily training was performed 6 days a week for 2–3 h each day. During a regular training period, different training sets were spread throughout a week due to time limitation. For example, the resistance training in the fitness room was held 4 days a week minimum, while the circuit and run trainings were only held twice a week. The fundamental training was held daily. The combination of position and team training was held for about 60 to 90 min every day. The team’s coaches created the training plan, and the researchers did not change their training schedule or its content. During the rest and regular training periods, neither food quality nor quantity were controlled. All of the subjects ate the same meals, including snacks; however, the quantity of their diets was not controlled, since they could ask for additional servings during the training camp. Body weight was monitored in the morning and before and after both training sessions. If there was more than 2% of weight loss, the consumption of water or other types of liquids was encouraged to avoid dehydration.

### 2.3. Anthropometry

On the morning of each test day, body weight was assessed using a digital body weight scale (UC-321, A&D Co. Ltd., Tokyo, Japan) after urination and under fasting conditions. Body height was measured once during the resting period to the nearest 0.1 cm using a stadiometer (YL-65, Yamagi, Inc., Nagoya, Japan).

### 2.4. Exercise Training Volume

To quantify the exercise volume of the intensive training camp, the validated session-rated perceived exertion (RPE) technique was used [20,21,22]. Intensity was assessed 30 min after each training session concluded using the RPE scale of Borg et al. [23]. During the regular training period, the duration and intensity of each training session were measured for 3 days to capture the weekly exercise training load. The exercise volume for each training day was obtained by multiplying the duration of training in hours by the intensity of each training session measured by RPE.

### 2.5. Dietary Intake

Carbohydrate, protein, and fat intakes were measured using 3-day dietary records. The subjects were instructed to record all food items and beverages consumed, and to provide detailed descriptions of each food item, including the location of purchase, weights, and proportions consumed. The subjects were also asked to provide photos of the food they consumed, including food labeling if available. Research dietitians interviewed the participants after they had completed the forms and analyzed the records using nutritional analysis software (Wellness21 Top Business System Co., Ltd., Okayama, Japan). Energy and nutrition intake were calculated based on the Japanese Standard Food Composition Table 2010 published by the Ministry of Education, Culture, Sports, Science, and Technology [24].

### 2.6. Hormonal and Biochemical Indicators

Blood indicators: Venous blood samples were collected in the morning after all other measurements were taken. Subjects were asked to fast for approximately 10 h, during which time only water was allowed. The blood samples were collected into ethylenediaminetetraacetic acid (EDTA)-aprotinin monovettes for acylated ghrelin and EDTA-2Na for the cortisol and insulin analyses. Whole blood samples were used to analyze glucose measurements, while the plasma was separated by centrifugation and aliquoted into storage tubes for the rest of the measurements. For the acylated ghrelin analysis, 100 µL of 1 M hydrochloric acid was added per milliliter of plasma to stabilize the substrate. The blood samples were kept either frozen or refrigerated appropriately, and were then analyzed by LSI Medience Corporation in Japan. The plasma cortisol and insulin levels were measured using a chemiluminescent immunoassay, the acylated ghrelin level was measured using enzyme-linked immunosorbent assay (ELISA), and the blood glucose level was measured using an enzymatic method. The inter-assay and intra-assay coefficients of variation for cortisol were 3.9–6.0% and 5.4–6.3%, respectively, for acylated ghrelin they were 4.7–8.1% and 2.1–8.3%, respectively, for insulin they were 1.9–2.9% and 2.5–3.9%, respectively, and for blood glucose they were 0.8–1.6% and 0.2–0.5%, respectively.

Salivary indicators: For the SIgA measurements, the subjects were asked to rinse out their mouths with sterilized water three times for 30 s and then wait for at least 5 min. Subjects chewed sterilized cotton (Salivette; Saersted, Vümbrecht, Germany) for 1 min at a frequency of 1 chew per second to stimulate saliva production. The SIgA concentration was measured using an ELISA. The SIgA secretion rate (µg∙min^−1^) was calculated by multiplying the SIgA concentration (μg∙mL^−1^) by the saliva flow rate (mL∙min^−1^), which was calculated by dividing the total volume of saliva in each sample (mL) by the time taken to produce the sample (min).

### 2.7. Appetite and Fatigue Sensation

A paper and pen–based visual analog scale (VAS) was used to assess sensations of hunger, fullness, and prospective food consumption, as well as fatigue, by asking the following questions: (1) How hungry do you feel? (2) How full do you feel? (3) How much do you think you can eat? (4) How tired are you? A 100 mm long VAS was created with words anchored at each end indicating the most positive and negative ratings. The subjects were not allowed to discuss or compare their ratings with each other, nor could they refer to their previous ratings. A translated and validated appetite sensation questionnaire was used in this study [25,26]. The VAS questionnaire of fatigue was also 100 mm in length, with the left end stating not tired at all and the right end marked as extreme exhaustion.

### 2.8. Mood States

The profile of mood states (POMS) scale is used to monitor changes in a negative psychological state as a stress reaction [2,27]. A validated Japanese translated POMS questionnaire (brief version) was administered in the early morning at the same time the appetite VAS questionnaire was conducted [28]. POMS provides measures of tension, depression, anger, vigor, fatigue, and confusion. The total mood score of the POMS was calculated by subtracting the vigor (positive) score from the sum of the five other (negative) measures and then adding 100 [29]. A higher global score means a more negative mood profile.

### 2.9. Statistical Analysis

The data are presented as the mean ± standard deviation (SD). The statistical analyses were performed with SPSS version 23.0 (SPSS, Inc., Chicago, IL, USA). A one-way analysis of variance (ANOVA) with repeated measures across time was used for the statistical evaluation of the data. When ANOVA revealed a significant main effect, post hoc analyses were performed using the Tukey procedure for equal variance and the Games–Howell procedure for unequal variance. Statistical significance was set at *p* < 0.05.

## 3. Results

### 3.1. Anthropometric Measures

The mean body height was 177.0 ± 4.7 cm. The mean body weights were 78.7 ± 6.8 kg, 77.9 ± 6.2 kg, 79.0 ± 7.0 kg, and 79.6 ± 7.1 kg for Rest, Regular training, Camp1, and Camp2, respectively. Body weight was lowest during a regular training period, and differed significantly from those of Camp1 (*p* = 0.012) and Camp2 (*p* = 0.001). There was no significant difference in body weight between Camp1 and Camp2.

### 3.2. Training Volume and Nutritional Intake

The ranges of temperature and humidity during the regular training period were 25–27 °C and 43–68%, respectively, while those of the training camp were 21–24 °C and 68–84%, respectively. The one-way ANOVA test revealed a significant difference in average perceived intensity between regular training at home and the start of training camp (Table 1). Moreover, the training duration at camp was approximately three times longer than during the regular training period. Therefore, the overall training volume per day during the training camp period was more than three times greater than that of the regular training period. There were no significant changes in training volume during the training camp.

Energy, carbohydrate, and fat intake were higher throughout the training camp period than during Rest or Regular training (Table 2). The overall intake of energy and all three nutrients significantly decreased toward the end of the training camp compared to at the start of the camp. The protein intake at the end of the training camp was approximately the same as that during the regular training period.

### 3.3. Measurements Related to Stress

**Figure 1:**
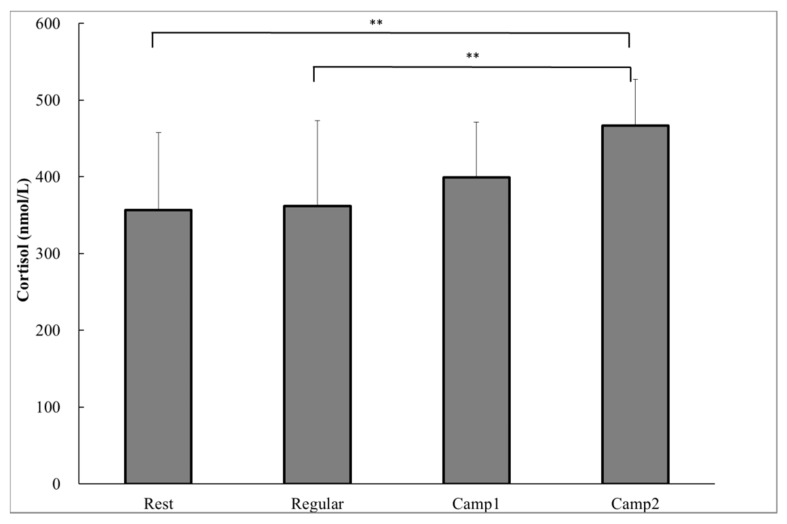
Plasma cortisol of power-trained athletes measured in the early morning during the rest, regular training, and intensive training camp periods. Data were analyzed using one-way analysis of variance with repeated measures. Values are shown as the mean ± standard deviation (SD) (*n* = 13). ** Significant difference at *p* < 0.01.

As a biochemical measure, the plasma cortisol concentrations were significantly higher at Camp2 (466.7 ± 60.7 nmol∙L^−1^) than at Rest (356.3 ± 100.9 nmol∙L^-1^; *p* = 0.002) or Regular training (361.7 ± 111.4 nmol∙L^−1^; *p* = 0.003; Figure 1). There was a significant decrease in saliva flow rate and a significant increase in SIgA concentration from Camp2 compared to Rest (saliva flow rate, *p* = 0.029; SIgA concentration, *p* = 0.039) or Regular training (saliva flow rate, *p* = 0.012; SIgA concentration, *p* = 0.024; Table 3). A correlation was found between the change in saliva flow rate and SIgA concentration from Regular training to Camp2 (*r* = −0.658, *p* = 0.028). Thus, the SIgA secretion rate did not differ significantly among the training or resting periods. None of the POMS measurements were significantly different between any of the periods (Table 4). During the training camp, whole body fatigue measured by VAS significantly increased from Rest or Regular (Table 5). There were no significant correlations between plasma resting cortisol and salivary indicators.

### 3.4. Measurements Related to Appetite 

**Figure 2:**
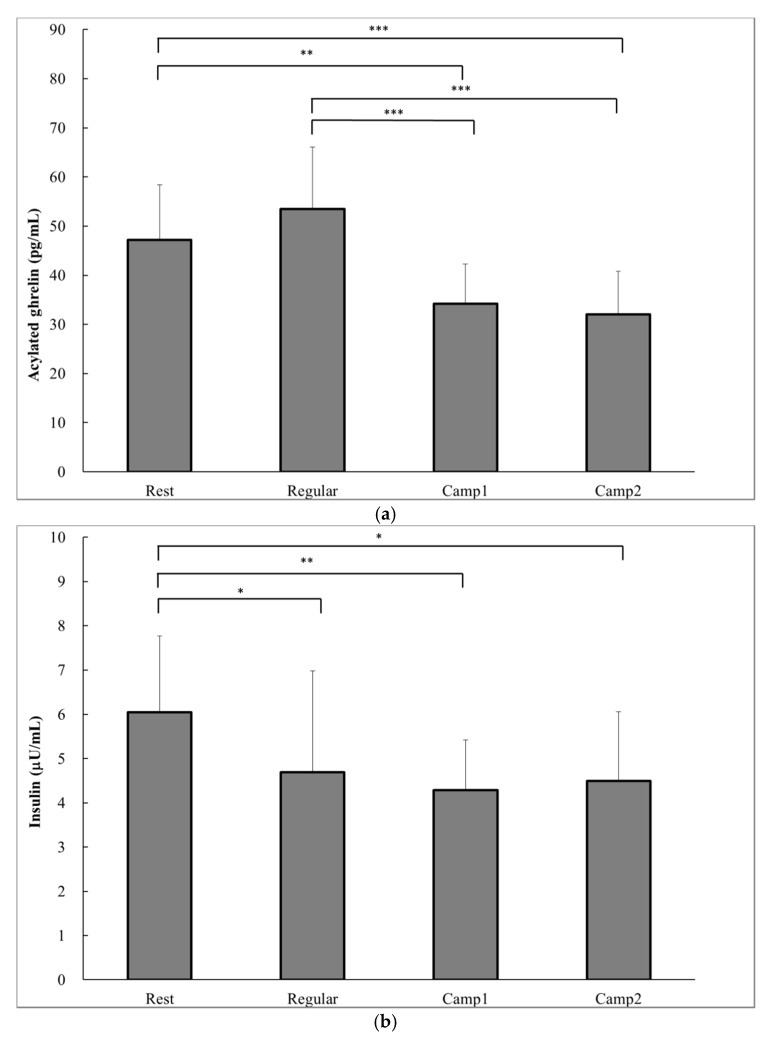

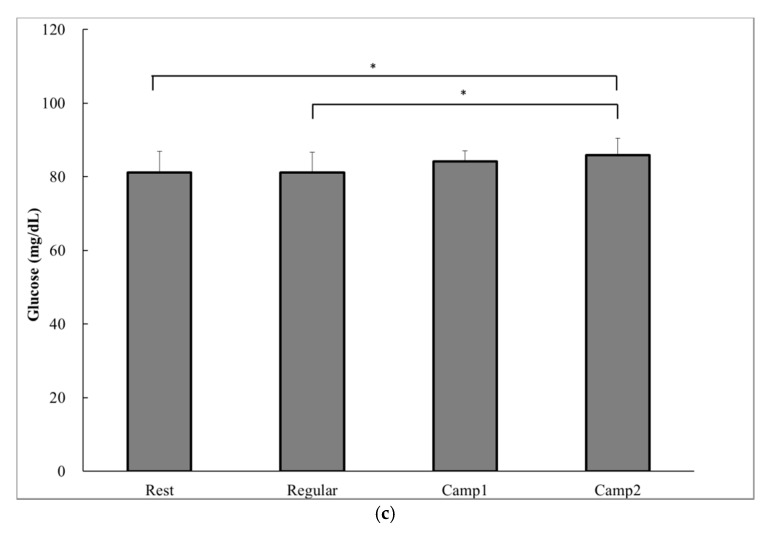
Hormonal and biochemical indicators of power-trained athletes at rest, regular training, and intensive training camp periods. (**a**) Acylated ghrelin; (**b**) Plasma insulin; (**c**) Glucose. Data are analyzed using a one-way analysis of variance (ANOVA) with repeated measures. Values are shown as the mean ± SD (*n* = 13). * Significant difference at *p* < 0.05; ** Significant difference at *p* < 0.01; *** Significant difference at *p* < 0.001.

According to a one-way ANOVA, a significant decrease in prospective food consumption was observed at Camp2 than at Rest (*p* = 0.014; Table 5). The acylated ghrelin level was significantly lower at both Camp1 (34.2 ± 8.0 pg∙mL^−1^) and Camp2 (32.0 ± 8.7 pg∙mL^−1^) than at Rest (47.2 ± 11.2 pg∙mL^−1^) or Regular training (53.4 ± 12.6 pg∙mL^−1^; Camp1 vs. Rest, *p* = 0.002; Figure 2a). The plasma insulin level significantly decreased from Rest (6.0 ± 1.7 µU∙mg^−1^) to Regular training (4.7 ± 2.3 µU∙mg^−1^, *p* = 0.04) and to training camp (Camp1: 1.3 ± 1.1 µU∙mg^−1^, *p* = 0.005; Camp2: 4.5 ± 1.6 µU∙mg^−1^, *p* = 0.015; Figure 2b). There were significant increases in blood glucose at the end of camp (Camp1: 84.2 ± 2.9 mg∙dL^−1^; Camp2: 85.8 ± 4.6 mg∙dL^−1^) compared with Rest (81.2 ± 5.7 mg∙dL^−1^, *p* = 0.028) and Regular training (81.2 ± 5.6 mg∙dL^−1^, *p* = 0.028; Figure 2c). There was no correlation between acylated ghrelin level and appetite sensations. Plasma acylated ghrelin level was negatively correlated with the change in cortisol concentrations from Regular training to Camp2 (*p* = 0.007) and from Camp1 to Camp2 (*p* = 0.04; Table 6).

## 4. Discussion

The current study was designed to investigate the early signs of changes in stress and appetite responses in male power-trained athletes during an intensive training camp. The primary finding of this study was that acute physiological training stress was observed at the end of the training camp, as evidenced by an increase in plasma resting cortisol concentration. In addition, the prospective and actual food consumption as well as the acylated ghrelin concentration decreased during the training camp. Moreover, due to decreased plasma resting cortisol and acylated ghrelin levels toward the end of the training camp, physiological training stress may have contributed to decreased acylated ghrelin secretion. We believe that this is the first study to observe the correlation between changes in appetite with acute physiological training stress in athletes during a shorter-duration training camp.

Since the training volume during training camp was more than three times as large as the regular training period, we speculated that the consecutive high-volume training placed a significant physical strain on the athletes. In fact, the fatigue sensation measured by VAS significantly increased during the training camp. Cortisol is the hormone secreted from the adrenal cortex in response to physiological stress, and it is commonly used as an early sign of training stress [30]. During prolonged excessive training, sympathetic activity is increased and cortisol secretion is stimulated at the acute phase of the stress responses [31]. Interestingly, we found that cortisol level significantly increased only at Day 9. It is possible that only one day of whole-day training before the Camp1 measurement did not cause enough physical strain to increase the cortisol level. In a study by Kirwan et al., cortisol concentrations at days 5 and 11 during intensive training were significantly increased [32]. Therefore, it is most likely that consecutive, high-volume training increased plasma resting cortisol levels at the end of training camp in this study. Immune function decreases with overtraining in athletes, as evidenced by a decrease in SIgA secretion rate [7,33]. However, no changes in SIgA secretion rate or mood state were observed in this study. Moreover, the POMS scores were stable and stayed relatively low compared to other consecutive intensive training studies [27,29]. Therefore, these findings suggest that the consecutive high-volume training during the 9-day camp induced acute physiological training stress, as seen in the increased resting plasma cortisol levels; however, the training stress severity was not high, as the immune function and psychological state were not influenced.

In this study, the subjective appetite sensation observed in the questions that assessed prospective food consumption decreased at the end of the training camp. Actual food intake was also significantly decreased from the beginning of the camp to the end of the training camp. Not only that, the concentration of acylated ghrelin declined during the training camp. There was no correlation between acylated ghrelin level and appetite sensations in the current study as seen in other studies [13]. Interestingly, there was a negative correlation between changes in cortisol and resting acylated ghrelin levels in the current analysis. Schmid et al. showed that a single intravenous injection of ghrelin stimulated hunger and increased cortisol concentration [34]. However, it was also reported that the sympathetic nervous system contributes to controlling ghrelin secretion. Hosoda and Kanagawa demonstrated that plasma acylated ghrelin concentration was influenced by alpha-adrenergic antagonists and beta-adrenergic agonists, which indicates that stomach ghrelin secretion is directly regulated by the sympathetic nervous system [35]. Increases in sympathetic activity are also known to decrease insulin secretion and increase glucose availability for the central nervous system, a finding that agrees with the results of this study [30]. Furthermore, an increase in sympathetic stimulation also reportedly decreases salivary flow rate [36]. Therefore, increased cortisol, increased blood glucose, and decreased insulin levels as well as salivary changes support the hypothesis that sympathetic nervous system activity increased during training camp, particularly toward the end. Thus, it is possible that increased sympathetic nervous activity contributed to the decreased acylated ghrelin level during the intensive training camp. Espelund et al. observed an inverse association between serum total ghrelin and serum cortisol levels during 48 h of fasting, although the underlying mechanisms are not clearly understood [37]. Unfortunately, since many factors may be involved in a change of appetite in this field study, further research is necessary to elucidate the physiological mechanisms behind the changes in an appetite regulatory hormone during an intensive training period in relation with stress reactions.

The limitation of this study is that there were no objective measures of training volume, such as % maximum oxygen uptake, % repetition maximum, or using an activity tracking device. Since American Football is a collision-based team sport, it is challenging to assess the influence of violent impact forces on training monitoring. Furthermore, it puts athletes at risk of injury to wear a hard device. Clarke et al. showed significant correlation between session-RPE and heart rate-based training load measures in another collision-based team sport [38]. Therefore, in this study, the exercise volume was measured using the session RPE technique to confirm that the exercise volume during training camp was significantly higher than that in the regular training period. The second limitation is that dietary intake was not controlled in this study. During the training camp, these athletes consumed large amounts of food to compensate for the energy demands of the prolonged training as well as to increase their muscle mass. Therefore, nutritional intake during training camp was quite high, particularly at the beginning. However, nutritional intake significantly decreased toward the end of training camp despite the training volume remaining the same. There was no change in acylated ghrelin concentration between Camp1 and Camp2. For these reasons, it is likely that changes in ghrelin were unrelated to hunger and food intake, but instead were probably associated with physiological training stress.

## 5. Conclusions

This study aimed to investigate appetite changes in relation to training stress in male power-trained athletes during a training camp to understand the cause of loss of appetite during strenuous training periods. As a result, although both acylated ghrelin and prospective food consumption decreased at the end of the training camp, no relationship was found between ghrelin and loss of appetite. Instead, this physiological training stress from the intensive training camp may have contributed to the decreased acylated ghrelin level. In conclusion, this study’s findings suggested that even the earlier phase of a physiological stress response to consecutive high-volume training during an intensive training camp might decrease the acylated ghrelin level in male power-trained athletes.

## Figures and Tables

**Table 1:** Training intensity, duration, and volume of power-trained athletes at regular training (average of 3 training days) and the start and end of training camp.

*n* = 13	Regular	Camp1	Camp2
RPE per training session	13.9 ± 0.9	15.0 ± 1.2 ^a^	14.8 ± 2.9
Total training duration (hours/day)	2.2 ± 0.4	7.1 ± 0.1 ^a^	6.8 ± 0.8 ^a^
Total training volume (RPE hour/day)	31.1 ± 5.9	106.9 ± 8.5 ^a^	99.5 ± 20.1 ^a^

^a^ Significant difference (*p* < 0.05) compared to Regular. RPE, rated perceived exertion.

**Table 2:** Daily intake of energy and major nutrients of power-trained athletes at rest, regular training, and intensive training camp periods.

*n* = 13	Rest	Regular	Camp1	Camp2
Energy (kcal)	3373 ± 769	3398 ± 739	6175 ± 513 ^a,b^	4752 ± 915 ^a,b,c^
Carbohydrate (g)	531 ± 128	485 ± 108	926 ± 108 ^a,b^	747 ± 196 ^a,b,c^
Protein (g)	105 ± 22	125 ± 27	180 ± 24 ^a,b^	134 ± 23 ^a,c^
Fat (g)	83 ± 31	97 ± 30	184 ± 26 ^a,b^	131 ± 32 ^a,b,c^

^a^ Significant difference (*p* < 0.05) compared to Rest; ^b^ Significant difference (*p* < 0.05) compared to Regular; ^c^ Significant difference (*p* < 0.05) compared to Camp1.

**Table 3:** Saliva flow rate, SIgA concentration, and SIgA secretion rate of power-trained athletes at rest, regular training (average 2 days), and intensive training camp periods.

*n* = 13	Rest	Regular	Camp1	Camp2
Saliva flow rate (mL/min)	1.1 ± 0.5	1.2 ± 0.5	1.1 ± 0.6	0.8 ± 0.6 ^a,b^
SIgA concentration (μg/mL)	24.6 ± 11.1	24.0 ± 13.8	26.6 ± 6.2	32.1 ± 9.0 ^a,b^
SIgA secretion rate (µg/min)	27.2 ± 16.1	25.9 ± 12.0	28.1 ± 13.3	24.3 ± 14.6

^a^ Significant difference (*p* < 0.05) compared to Rest; ^b^ Significant difference (*p* < 0.05) compared to Regular.

**Table 4:** The profile of mood states (POMS) scores of power-trained athletes at rest, regular training (average 2 days), and intensive training camp periods.

*n* = 13	Rest	Regular	Camp1	Camp2
Tension	1.5 ± 4.4	1.3 ± 2.2	1.4 ± 1.7	2.2 ± 3.2
Depression	1.5 ± 3.2	1.0 ± 1.6	0.8 ± 1.4	1.2 ± 2.1
Anger	0.6 ± 1.6	0.8 ± 1.3	0.8 ± 1.5	0.7 ± 1.3
Vigor	1.0 ± 1.7	2.4 ± 2.0	1.4 ± 2.7	1.6 ± 3.6
Fatigue	3.1 ± 4.3	4.2 ± 4.7	4.6 ± 3.6	6.5 ± 5.2
Confusion	1.5 ± 3.0	1.1 ± 1.6	0.5 ± 1.0	1.2 ± 2.2
Total mood score	107.2 ± 14.9	105.9 ± 9.6	106.8 ± 5.6	110.2 ± 10.6

**Table 5:** Appetite and fatigue sensation scores of power-trained athletes at rest, regular training (average 2 days), and intensive training camp periods.

*n* = 13	Rest	Regular	Camp1	Camp2
How hungry do you feel?	67 ± 20	60 ± 18	50 ± 18	60 ± 17
How full do you feel?	31 ± 22	29 ± 19	42 ± 22	40 ± 21
How much do you think you can eat?	62 ± 15	55 ± 15	51 ± 14	47 ± 15 ^a^
How tired are you?	42 ± 14	49 ± 16	66 ± 11 ^a,b^	77 ± 15 ^a,b^

^a^ Significant difference (*p* < 0.05) compared to Rest; ^b^ Significant difference (*p* < 0.05) compared to Regular.

**Table 6:** Correlation coefficients (*r*) and probability values (*p*) related to the ∆ plasma resting cortisol concentration and ∆ Acylated ghrelin, ∆ Insulin, and ∆ Glucose levels with a combination of Rest or Regular training periods between training camp periods.

	∆ Acylated Ghrelin		∆ Insulin		∆ Glucose	
∆ Cortisol	*r*	*p*	*r*	*p*	*r*	*p*
∆ Rest to Camp2	−0.365	0.220	−0.510	0.075	0.147	0.631
∆ Regular to Camp2	−0.705	0.007 *	−0.136	0.657	0.109	0.722
∆ Camp1 to Camp2	−0.575	0.040 *	−0.213	0.485	0.289	0.339

* Significant difference at *p* < 0.05.
